# Some Properties of the *C. elegans* Multicopper Oxidase F21D5.3, an Ortholog of Human Ceruloplasmin

**DOI:** 10.3390/ijms26104776

**Published:** 2025-05-16

**Authors:** Polina D. Samuseva, Aleksandra A. Mekhova-Caramalac, Federico Catalano, Anna D. Shchukina, Sofia A. Baikina, Daria N. Magazenkova, Ludmila V. Puchkova, Ekaterina Yu. Ilyechova

**Affiliations:** 1 Research Center of Advanced Functional Materials and Laser Communication Systems, ADTS Institute, ITMO University, 197101 St. Petersburg, Russia; 2 Laboratory of Biochemical Genetics, Research Institute of Experimental Medicine, 197022 St. Petersburg, Russia; 3 Institute of Biomedical Systems and Biotechnology, Peter the Great St. Petersburg Polytechnic University, 195251 St. Petersburg, Russia; 4 Institute of Biosciences and BioResources, National Research Council (CNR), 80131 Naples, Italy

**Keywords:** *C. elegans*, worm ceruloplasmin homolog F21D5.3, Wilson’s disease, worm model of Wilson’s disease, *cua-1* gene, silver nanoparticles

## Abstract

This study identified an oxidase-positive protein in the plasma membrane fraction of the *C. elegans* N2 strain. The protein with a molecular weight of approximately 85 kDa reacted with antibodies against human and mouse, but not rat, ceruloplasmin and exhibited oxidase activity. Bioinformatic analysis revealed that the F21D5.3 protein possesses four copper-binding sites, similar to those in other multicopper oxidases (MCOs), and plastocyanin-like domains characteristic of MCOs. However, neither an iron-binding domain nor ferroxidase activity, typical features of MCOs, were detected through in silico analysis and or in-gel assays. Despite the absence of ferroxidase activity, these findings suggest that the protein may be the product of the *F21D5.3* gene, an ortholog of MCOs in *C. elegans*. Heat map analysis indicated *F21D5.3* expression in the entero-rectal valve cells and both the anterior and posterior intestines. Among the genes associated with copper transport, only *cua-1* exhibited a similar expression pattern. In the *C. elegans cua-1^H828Q^* strain, which mimics a mutation in human ATP7B linked to Wilson’s disease (WD), oxidase activity was also observed. Notably, both strains showed reduced oxidase activity when cultured with silver nanoparticles (AgNPs). These findings highlight the potential of the *cua-1^H828Q^* strain as a model for studying copper and iron metabolism and for developing therapeutic strategies for WD.

## 1. Introduction

Copper is an essential element for almost all living organisms, participating in antioxidant protection, photosynthesis, and oxidative phosphorylation. The biological functions of copper became increasingly diverse as organisms evolved and became more complex. Currently, in mammals, copper serves as a catalytic factor for approximately two dozen enzymes that control fundamental cellular processes, such as respiration, neutralization of ROS, formation of connective tissue, synthesis and catabolism of neurotransmitters, maturation of neuropeptides, transport of iron across biological membranes, degradation of volatile sulfur compounds, and others [1,2]. In addition, copper ions, as regulators of signaling pathways, neurotransmission, apoptosis, and the activity of several transcription factors, control the overall metabolic state of the cells [2].

In a biological environment, “free” copper ions catalyze the generation of ROS [3]. The conservative system of copper homeodynamics protects cells from this potential danger, ensuring the safe transfer of copper from the extracellular environment to copper-dependent protein formation and operation sites [4]. Hence, it is not surprising that inborn errors of copper metabolism lead to the development of severe, socially burdensome neurodegenerative, oncological, and metabolic diseases including Wilson’s disease (WD), Menkes disease, aceruloplasminemia, MEDNIK syndrome, amyotrophic lateral sclerosis, some forms of Parkinson’s disease and Alzheimer’s disease, common cancers, type 2 diabetes, obesity, hypertension, and others [5,6,7,8,9,10,11,12,13,14]. The effectiveness of their treatment depends directly on the identification of the copper metabolism links responsible for the shifts in copper homeostasis and the successful search for approaches to influence them. Most of the research in this area has been conducted on cultured human cells [15,16] and/or model laboratory mammals with defects in copper metabolism genes [17,18,19]. However, both models have certain limitations. Specifically, cultured cells do not receive signals from cells of other organs, so proteins whose expression is regulated in a cross-talking manner do not function in cultured cells in vitro, such as copper-dependent thiol oxidase [20]. On the other hand, mammals have a very complex system that controls copper homeostasis, and many of its participants remain unknown.

These general limitations are absent in *C. elegans*, whose copper homeostasis system is simpler than that of mammals. Nevertheless, it possesses major structural and functional orthologs of human copper-regulatory proteins [21]. As a result, *C. elegans* is increasingly used as a model organism for studying copper-associated diseases. The most common among them is WD—an autosomal recessive genetic disorder in humans caused by mutations in the *ATP7B* gene, which encodes a copper-transporting ATPase responsible for incorporating copper into ceruloplasmin (Cp, the major copper-carrying protein in the blood) and exporting excess copper into bile. When ATP7B function is impaired, copper accumulates first in the liver and/or brain, leading to severe hepatic and neurological complications that can ultimately be fatal [22].

The orthologue of the human copper-transporting ATPase ATP7B in *C. elegans* is CUA-1, which shares approximately 45% sequence identity with human ATP7B [23]. In this context, a *C. elegans* line carrying the H828Q substitution in the CUA-1 protein was developed [24]. This mutation corresponds to the homologous H1069Q mutation in human ATP7B, which is found in more than 50% of patients [25]. It has been shown that *cua-1^H828Q^* nematodes are sensitive to copper overload, similar to WD patients, and pharmacological chelators reduce the toxic effect of copper. In addition, these nematodes exhibit signs of neurodegeneration. These findings strongly support the role of CUA-1 in copper excretion. However, it remains unclear whether CUA-1, like ATP7B, is involved in the metallation of copper-dependent enzymes within the Golgi apparatus, and whether the H828Q substitution impairs this function. In the present study, the expression of the *F21D5.3* gene and some properties of its putative protein product, F21D5.3, were examined in both wild-type and the *cua-1^H828Q^* strain nematodes.

## 2. Results

### 2.1. Description of the Putative Cp-like Protein F21D5.3

**Figure 1:**
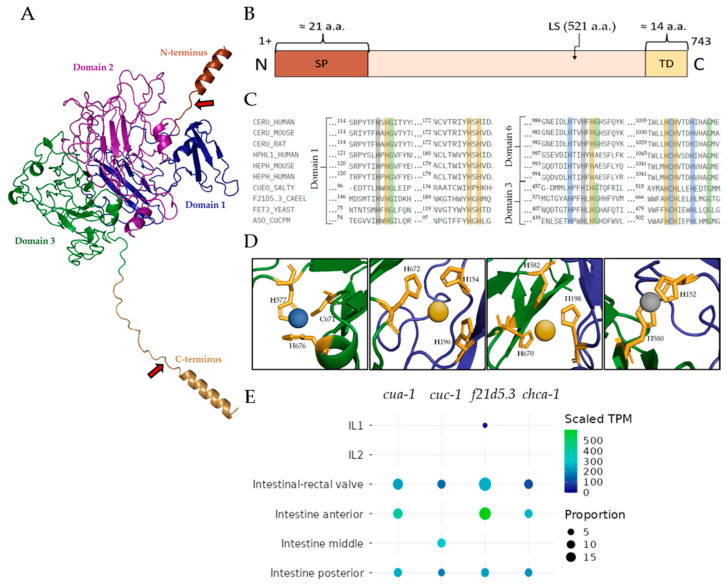
(**A**) Putative spatial model of F21D5.3, including a *C*-terminal GPI-anchor addition site (predicted using D-I-TASSER URL: https://zhanggroup.org/, accessed on: 13 January 2025); plastocyanin-like domains are colored blue, magenta and green. (**B**) Domain arrangement of F21D5.3. The N-terminal domain is depicted in red, the C-terminal transmembrane domain is depicted in yellow, and the mature protein is depicted in pink; LS—labile site. (**C**) Alignment of plastocyanin-like domains of MCOs from different phylogenetic groups (trivial species names, gene names, and sources of amino acid sequences are provided in Table S2). (**D**) Structures of the respective copper-binding centers in the predicted structure. The mandatory MCO copper-binding residues are shown, and putative positions of copper ions are indicated. The colors of residues in section C and copper ions in section D correspond, in addition to copper center types (blue for type 1 Cu, grey for type 2, yellow for type 3). Color of the polypeptide chain in section C is the same as in section A. (**E**) Heat map of *F21D5.3* gene expression in *C. elegans* and expression of genes involved in copper transport to the Golgi complex lumen, homologous to the mammalian Cp metallation axis.

According to the in silico genome analysis of *C. elegans*, the *F21D5.3* gene is a worm ortholog of mammalian MCO genes. The putative protein product of this gene consists of 743 amino acid (aa) residues with a molecular mass of 85 kDa. The protein contains a labile site at which it can be cleaved into 60 kDa and 25 kDa fragments. The predicted tertiary structure and domain arrangement of F21D5.3 are shown in Figure 1A,B. At the *N*-terminus, it has a co-translationally cleavable 21-aa signal peptide and a 14-aa C-terminal alpha-helix, which could serve as a site for the addition of a glycosylphosphatidylinositol anchor (GPI anchor), judging by its properties (short transmembrane C-terminal domain). Five predicted N-glycosylation motifs (NXS/T) are present in F21D5.3 (N88, N217, N457, N534 and N597), but only the N217 site of the nematode protein corresponds to the N339 site of human Cp (Figure S1). The D-I-TASSER algorithm predicts three plastocyanin-like domains in the F21D5.3 structure, characteristic of MCO family proteins, with invariant amino acid residues involved in the formation of copper atom coordination spheres (Figure 1C,D). In evolution, MCOs are believed to originate from the duplication of the ancestral β-fold cupredoxin domain, containing a single «blue» Type I copper-binding center (T1Cu, formed by tightly coordinated Cys and 2His residues and a variable loose axial ligand, which is often but not necessarily a Met), with a new oxygen-reducing trinuclear copper cluster evolving at the interface of two of these domains [26]. Some ancestral cupredoxin domains may have lost their T1Cu through evolution, e.g., in mammalian MCOs, which possess six domains; functional T1Cu is present in domains 2, 4 and 6, with a trinuclear cluster between domains 1 and 6 [26]. The trinuclear cluster, comprising a single Type 2 copper center plus a pair of Type 3 copper centers, and one T1Cu are required for oxidase activity. The analysis of sequence similarity and the structure predicted by the D-I-TASSER algorithm reveals a conserved pattern of His residues in domains 1 and 3 (Figure 1C). A putative T1Cu site in domain 3 is similar to that in domain 6 of mammalian Cp. It strongly suggests the presence of a trinuclear copper cluster between domains 1 and 3, and an adjacent T1Cu in domain 3. Therefore, the putative domains 1 and 3 of F21D5.3 are highly analogous to domains 1 and 6 of Cp, respectively, and can form a functional oxidase.

The putative copper-binding sites of F21D5.3 are almost identical to those of MCOs from other organisms. The BLASTP program revealed the highest similarity in the primary structure of F21D5.3 with human (>32%) and mouse (>34%) Cp, and yeast ferroxidase Fet3 (>29%) (Figure S2). A targeted search did not reveal an iron-binding site in F21D5.3, although this is a specific characteristic of mammalian MCOs and Fet3. F21D5.3 gene expression analysis and its co-localization with transcripts of other copper transports using heat mapping showed that the gene was expressed in a descending order in entero-rectal valves, as well as anterior and posterior parts of the intestine (Figure 1E). The *F21D5.3* gene is not expressed in the cells of the middle part of the intestine. Among all of the tested genes encoding copper transporters, only *cua-1* demonstrated both complete co-expression and transcripts per million values coincident with the *F21D5.3* gene (Figure 1E).

### 2.2. Effect of Silver Nanoparticle Treatment on the Worm Lifespan

Silver ions (Ag^+^), which gradually dissociate from the surface of AgNPs, can enter the body of various organisms, interfere with the Cu(I) transport system, and be delivered to the sites of cuproenzyme formation [27]. Silver can occupy sites in cuproenzyme coordination spheres that include cysteine or methionine residues, resulting in the loss of enzyme activity. Such effects have been reported for laccase [28], rat and mouse Cp [29,30], and phenol oxidase in mollusks [31]. To study the properties of F21D5.3, AgNPs were used as a tool to trace the pathway of copper incorporation into the active sites of nematode MCOs. AgNPs offer a reproducible source of silver ions, maintaining low yet stable concentrations of bioavailable silver over extended periods of worm development. In contrast, silver salts such as AgNO_3_ would provide a short-term, high-concentration exposure, which may not accurately reflect the slower and more complex processes associated with nanoparticle exposure. Therefore, AgNPs were chosen to better represent these interactions and more closely mimic real-world environmental exposures.

**Figure 2:**
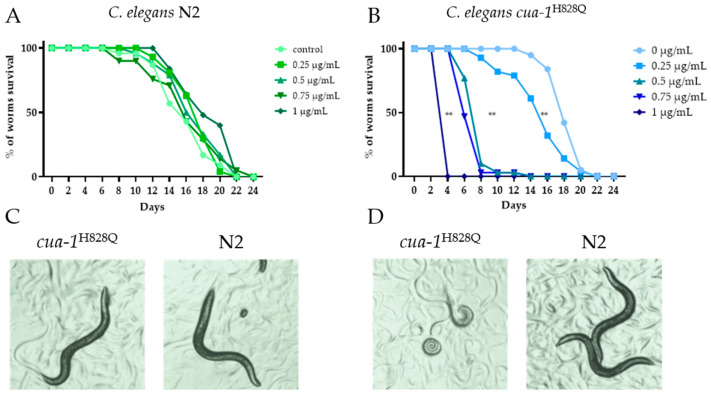
The effect of AgNPs on the lifespan of wild-type and *cua-1^H828Q^* nematodes. Survival curves of (**A**) N2 wild-type and (**B**) *cua-1^H828Q^* strains cultured in the presence of different concentrations of AgNPs. (**C**) Worms were cultured in the presence of 0.25 μg/mL of AgNPs; (**D**) worms were cultured in the presence of 1.0 μg/mL of AgNPs. The data were subjected to Log-rank test (Mantel-Cox): (**)—*p*-value < 0.01.

Initially, a concentration of AgNPs below that causing toxicity but sufficient to observe the effect of Ag^+^ was determined. For this purpose, a lifespan test was performed. The results, presented in Figure 2, show that the sensitivity threshold of *cua-1^H828Q^* nematodes to AgNPs is much lower than that of wild-type nematodes (Figure 2A,B).

This difference is also highlighted by the images of worms cultured at various AgNP concentrations (Figure 2C,D). There was no obvious visual difference between the N2 and *cua-1^H828Q^* strains at 0.25 μg AgNPs/mL (Figure 2C). Wild-type worms cultured at an AgNP concentration of 0.25 μg/mL (Figure 2C) were visually indistinguishable from those cultured at 1.0 μg AgNPs/mL (Figure 2D). Meanwhile cultivation of the *cua-1^H828Q^* strain at 1.0 μg/mL proved fatal. Therefore, in the following experiments, the subtoxic AgNPs concentration of 0.25 μg AgNPs/mL was used.

### 2.3. The F21D5.3 Gene Expression During the Life Cycle of N2 and cua-1^H828Q^ Strains

**Figure 3:**
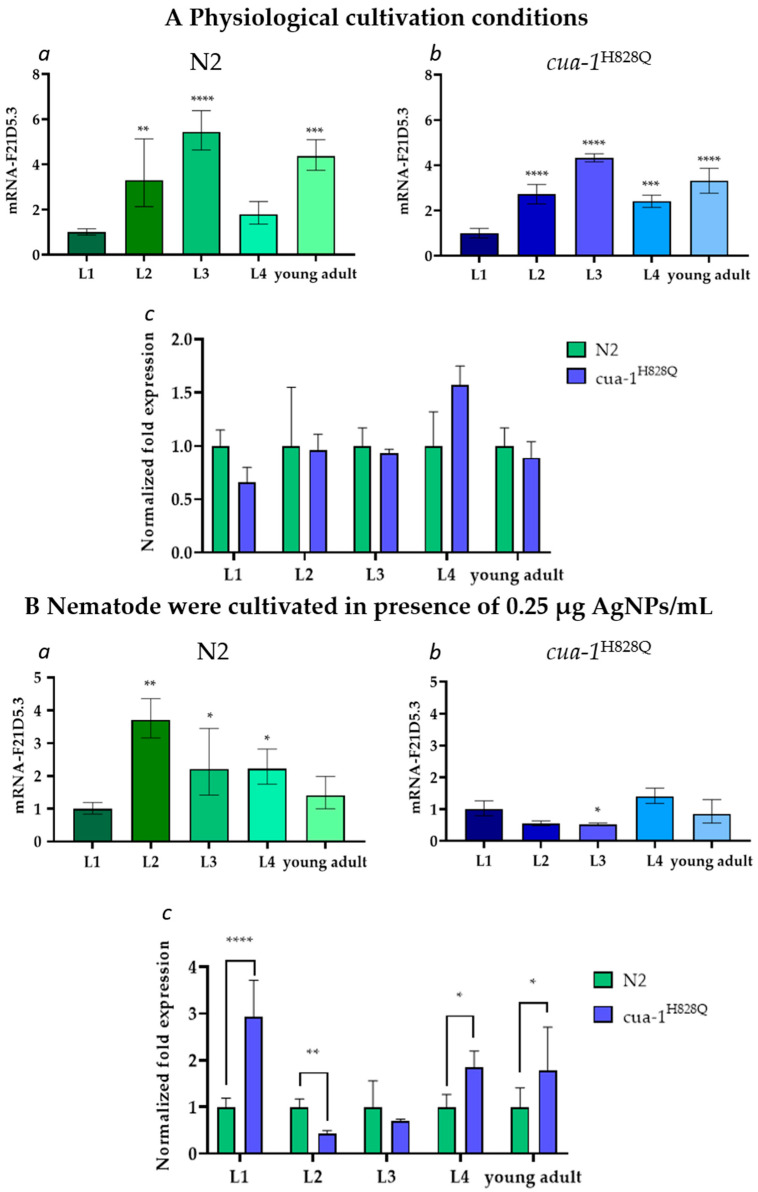
Changes in the content of mature transcription products of the F21D5.3 gene during the nematode life cycle under (**A**) physiological conditions and (**B**) in the presence of 0.25 μg/mL AgNPs. L1–L4: larval stages of the nematode; y.ad: young adult stage. *Y*-axis: concentration of F21D5.3 mRNA, a.u. (**a**,**b**)—F21D5.3 gene expression in N2 and *cua-1^H828Q^* strains under physiological conditions or in presence AgNPs, respectively; (**c**) normalized expression level of the *F21D5.3* gene in N2 and *cua-1^H828Q^* strains. The data were subjected to one-way ANOVA followed by Tukey’s post hoc test: (*)—*p*-value < 0.05; (**)—*p*-value < 0.01; (***)—*p*-value < 0.001; (****)—*p*-value < 0.0001).

Under physiological cultivation conditions, the *F21D5.3* gene is expressed similarly in both nematode strains at all stages of the life cycle (Figure 3(Aa,Ab)). However, its activity changes during ontogenetic development. In both strains, the F21D5.3 mRNA level increased approximately fivefold from the L1 to L3 stage, then dropped at the L4 stage and slightly increased at the young adult stage. No significant difference in the gene activity level was found between the N2 and *cua-1^H828Q^* strains (Figure 3Ac). There were also no differences in body morphology, size, or mobility that could be visually detected between wild-type and mutant individuals (Figure 2). At the same time, in wild-type nematodes cultured in the presence of AgNPs, the *F21D5.3* gene activity begin to decrease after the L2 stage (Figure 3Ba). In *cua-1^H828Q^* nematodes, the *F21D5.3* gene activity remained low throughout ontogenetic development (Figure 3Bb). Comparative analysis of F21D5.3 mRNA transcripts between N2 and *cua-1^H828Q^* strains revealed strong gene expression upregulation at the L1, L4, and young adult stages in the mutants (Figure 3Bc).

### 2.4. The Search for Antigenic, Oxidase, and Ferroxidase Activities in the Plasma Membrane Proteins of N2 and cua-1^H828Q^ Strains

**Figure 4:**
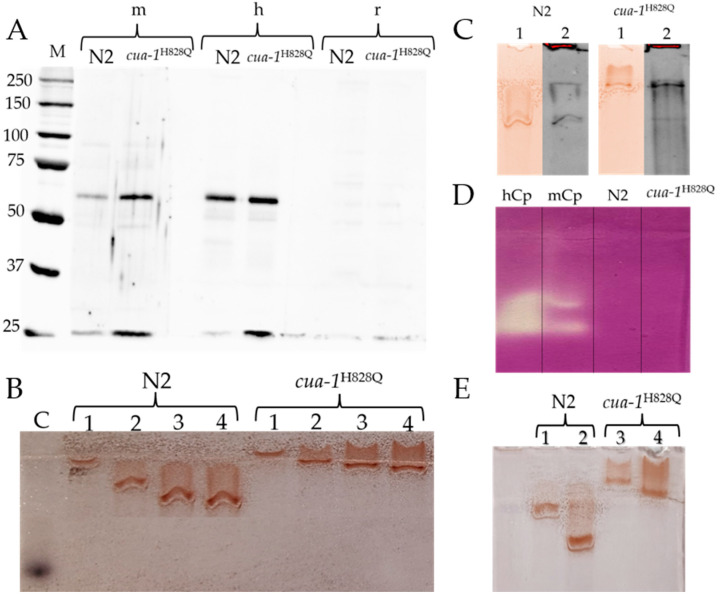
The putative protein product of the F21D5.3 gene (**A**) cross-reacts with antibodies to mouse, human, but not rat Cp, (**B**,**C**) exhibits oxidase, (**D**) but not ferroxidase activities, and (**E**) is sensitive to AgNP treatment. (**A**) A total of 30 μg of plasma membrane protein *per* lane was fractionated in 8% SDS-PAAG and analyzed by WB; “m”, “h”, and “r” represent antibodies to mouse, human, and rat Cp, respectively; M—protein molecular weight marker. (**B**) Aliquots of plasma membrane protein were fractionated in 6% PAAG under non-denaturing conditions, the gel was stained with o-dianisidine, a specific chromogenic substrate for MCOs. C (control)—2 μL of murine blood serum applied to the lane; 1–4: protein concentration of 5, 10, 15, 20 μg, respectively. (**C**) Plasma membrane proteins were fractionated on 8% PAAG under non-denaturing conditions, stained with (1) *o*-dianisidine, or (2) analyzed by WB. (**D**) Samples were fractionated in 8% PAAG in non-denaturing conditions, and the gel was stained for ferroxidase activity; 1: human Cp, 7 μg per lane; 2: mouse serum, 2 μL (approximately 0.25 μg of mouse Cp); 3 and 4: 45 μg plasma membrane protein per lane isolated from nematodes N2 and cua-1^H828Q^, respectively. (**E**) 1 and 2—N2 strain; 3 and 4—*cua-1^H828Q^*; 1 and 3—0.25 μg/mL AgNPs were added to worm culture medium; 2 and 4—untreated. Each sample contained 20 µg of protein.

Based on the high degree of identity of F21D5.3 with human and mouse Cp, we hypothesized that these proteins share identical epitopes, to which antibodies could be produced. To test this possibility, plasma membrane proteins were immunoblotted with antibodies to mouse, human, and rat Cp (Figure 4A). Antibodies to rat Cp did not reveal any immunoreactive polypeptides in these proteins. However, in the same samples, antibodies to human and mouse Cp revealed two fragments with molecular weights of approximately 62 kDa and 25 kDa. This is more than expected because F21D5.3 presented on the membrane is a mature protein that does not contain a signal peptide and C-terminal transmembrane domain, the total molecular mass of which is approximately 4.2 kDa. This difference is sufficient for its detection by electrophoresis in PAAG under denaturing conditions. However, since mature F21D5.3 contains a covalently bound GPI anchor, the minimum size of which is about 1.5 kDa, and the presence of a glycolipid component slows down the mobility of the polypeptide in the gel, it can be concluded that the detected fragments are entirely consistent in size with putative F21D5.3. The sum of these fragments corresponds to the putative Cp-like protein F21D5.3, and their size matches the mass of polypeptides that could presumably be formed upon cleavage of F21D5.3 at the labile site.

**Figure 5:**
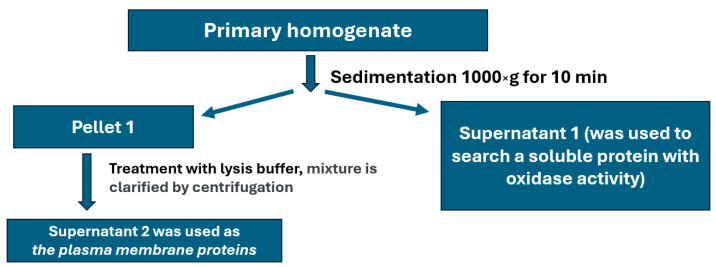
Scheme for isolating a fraction enriched with plasma membrane proteins.

Non-denaturing electrophoresis of plasma membrane protein samples, followed by *o*-dianisidine staining of the gel, revealed oxidase-positive protein bands (Figure 4B). Their intensity was proportional to the protein concentration in the sample. The slow and abnormal concentration-dependent movement mobility of the oxidase-active protein in non-denaturing conditions is due to the presence of Triton *X*-100 micelles in the sample. In both strains, the relative content of protein exhibiting oxidase activity was the same. In the gel, the positions of the zones stained for oxidase activity and those bound by antibodies to human Cp coincided (Figure 4C). No ferroxidase activity was detected in this protein fraction by the conventional method (Figure 4D). In Supernatant 1 (Figure 5), no oxidase activity specific to MCOs was found.

In both strains, the cultivation of nematodes in the presence of AgNPs resulted in a decrease in the oxidase-positive signal (Figure 4E). This correlates well with the decrease in the concentration of mRNA coding for F21D5.3 (Figure 3(Ba,Bb)). However, unlike in mammals [32], the cultivation of nematodes in the presence of AgNPs did not lead to full suppression of oxidase activity (Figure 4E). This may reflect differences in the intracellular routes of Cu(I) and/or Ag(I) in nematodes compared to mammals.

## 3. Discussion

Interest in *C. elegans* models with copper dyshomeostasis is increasing due to the widespread prevalence of copper-associated diseases in humans. In a significant cohort of such patients, Cp deficiency is the biochemical manifestation of copper dyshomeostasis. Although the molecular genetic causes of Cp deficiency differ in WD, aceruloplasminemia, Alzheimer’s disease, and Parkinson’s disease, the Cp level can be a valuable diagnostic and prognostic marker of copper imbalance.

In the present study, the properties of the putative nematode protein F21D5.3, an ortholog of human Cp, are demonstrated for the first time. The *F21D5.3* gene product was detected in the protein fraction extracted with Triton X-100 from plasma membranes. It was detected by immunoblotting with antibodies to human and mouse Cp, but not to rat Cp (Figure 4A). Interestingly, the same antibodies cross-react with mouse and human Cp but not rat Cp [33]. The protein exhibits MCO-specific oxidase activity toward aromatic amines but does not display ferroxidase activity, which is characteristic of Cp (Figure 4B,D). Importantly, the immunoreactive and oxidase-positive zones of this protein coincide in terms of electrophoretic mobility (Figure 4C). We cannot explain why F21D5.3 extracted from the *cua-1^H828Q^* plasma membrane migrates more slowly than F21D5.3 from the wild-type strain. However, this phenomenon was consistently observed in all experiments using non-denaturing PAGE (Figure 4B,C,E). One possible explanation is that the C-terminal transmembrane domain (TMD) is not cleaved in the mutant strain; however, this assumption requires further verification.

The experimental data are fully consistent with the bioinformatic analysis, which shows that the F21D5.3 protein belongs to the MCOs family: it possesses conserved copper binding sites identical to those of mammalian Cp. Additionally, F21D5.3 exhibits a high degree of identity with human and mouse Cp and contains sites for attachment to the plasma membrane via a GPI anchor. However, it lacks the iron-binding site characteristic of ferroxidases. Recently, it was shown that nematodes with an *F21D5.3* gene knockout develop features of impaired iron metabolism [34], suggesting that F21D5.3 is involved in iron metabolism. This is consistent with data demonstrating that the loss of F21D5.3 function results in increased levels of labile copper, induction of oxidative stress, and neurodegenerative-type behavior [35]. This discrepancy regarding the absence of an iron-binding site in F21D5.3 may indicate features of nematode iron metabolism in which the Cp ortholog plays an indirect role. Alternatively, F21D5.3 may be a weakly associated subunit of a ferroxidase complex, and thus the ferroxidase activity was not detected electrophoretically, or F21D5.3 may require a partner localized to the plasma membrane to exhibit ferroxidase activity. The *F21D5.3* gene is expressed throughout the nematode life cycle (Figure 3A). Its activity increases during ontogenesis, reaches a maximum at the L3 stage, and decreases during aging. The expression of *F21D5.3* in *cua-1^H828Q^* nematodes does not differ from that in wild-type worms at either the transcription or translation levels (Figure 3B and Figure 4A). However, treatment with AgNPs reveals that the sensitivity of the *cua-1^H828Q^* strain is significantly higher than that of the N2 strain (Figure 2). The concentration of AgNPs that does not cause external signs of poisoning in both strains suppresses the formation of mature transcripts of the *F21D5.3* gene and reduces the level of oxidase F21D5.3 (Figure 3B and Figure 4E). The decrease in oxidase activity may occur both due to the suppression of the *F21D5.3* gene activity observed when nematodes were treated with AgNPs (Figure 3) or because of the replacement of copper atoms with silver atoms during the metallation of F21D5.3.

Additional studies are needed to elucidate why patients with WD who have a completely normal *Cp* gene already exhibit Cp deficiency at the early stages of disease progression. The *C. elegans* presents a promising model for investigating the molecular mechanisms underlying Cp deficiency. Understanding how Cp orthologs function and fail in *C. elegans* may help illuminate early disturbances in copper homeostasis that precede clinical symptoms in human copper-associated diseases.

The wild-type and *cua-1^H828Q^* mutant strain of *C. elegans* may also be developed into powerful tool for studying the effects of long-term silver exposure, including AgNPs. The distinct response of F21D5.3 to AgNP exposure both in terms of gene expression and enzymatic activity levels reflects the complex interaction between copper metabolism and environmental stressors. This offers new opportunities to use *C. elegans* for exploring environmental toxicants that affect metal homeostasis and for dissecting the roles of MCOs in neurodegenerative processes.

Our findings provide valuable insights for researchers studying copper-associated disorders in humans and those employing *C. elegans* as a model system. They also support the further development of *C. elegans* as a translational model to advance our understanding of copper dyshomeostasis and its broader implications for human health.

## 4. Materials and Methods

### 4.1. Worm Strains and Cultivation Conditions

*C. elegans* were cultured at 20 °C on nematode growth medium (NGM) Petri plates seeded with *Escherichia coli* strain OP50 as a food source for general maintenance. Worms were transferred to a fresh plate every 5 days to avoid starvation. In this study, two strains of *C. elegans* were used: the Bristol N2 wild-type strain and the mutant *cua-1(knu781[H828Q])* strain, abbreviated as *cua-1^H828Q^* hereafter. *C. elegans* strains were obtained from the Institute of Biosciences and Bioresources - Consiglio Nazionale delle Ricerche (IBBR-CNR, Naples, Italy). The characteristics of the *cua-1^H828Q^* strain were described previously [23].

### 4.2. Synchronization of Nematodes for Liquid Conditions

To synchronize the nematodes, 15 adult worms were placed on NGM plates and incubated for 1 h at 20 °C. During this time each nematode laid about 50 eggs, and then adult individuals were removed. The obtained plates were incubated at 20 °C for 3 days. Afterward, the adult nematodes were washed off the plates using M9 buffer, centrifuged at 1500× *g* for 3 min, and washed several times again with M9 buffer to remove residual bacteria. The pellets were carefully collected. Two volumes of 15% sodium hypochlorite and 1 volume of 5M NaOH were mixed, and 1.5 mL of the resulting mixture was added to the samples. The samples were then shaken continuously for 5 min, until free-floating eggs were observed under a microscope. Sterile M9 buffer was added to samples to a total volume of 15 mL to stop the reaction. The samples were centrifuged at 3000× *g* for 3 min to precipitate the eggs; this was followed by the pellets being washed several times with M9 buffer. Finally, the supernatants were discarded, the pellets were resuspended, and the eggs were transferred to a liquid culture medium.

### 4.3. Worm Cultivation in Liquid Culture

On the first day, the hatching nematodes were incubated without *E. coli* OP50, resulting in developmental arrest at the first larval stage, L1, for synchronization. Synchronized populations of N2 and *cua-1^H828Q^* worms were grown in liquid culture with *E. coli* OP50. Tightly closed 300 mL flasks containing liquid medium (not more than 150 mL) were placed on a shaker (110 rpm) and incubated at 20 °C. Nematodes were subsequently collected at different stages of their life cycle (L1, L2, L3, L4, young adult) and washed several times with M9 buffer to remove bacteria; then, 200 μL of each worm sediment was transferred into 2 mL tubes. Three biological replicates were prepared for each experimental group.

### 4.4. Silver Nanoparticles (AgNPs) Characteristics

The AgNPs used in this study were previously produced and characterized [29,32]. Briefly, AgNPs were fabricated by reducing silver from an AgNO_3_ solution with hydrazine hydrate in micelles formed by potassium oleate. According to the UV/Vis absorption spectra, transmission electron microscopy, laser diffractometry, *X*-ray diffractometry, electron diffraction of the AgNPs in the selected area, and the AgNPs were characterized by a high purity of crystals, their spherical form, and a size range of 30–40 nm. TEM studies were performed using equipment of the Federal Joint Research Center “Material science and characterization in advanced technology” supported by the Ministry of Science and Higher Education of the Russian Federation. The fresh aqueous suspensions of AgNPs contained only crystalline silver with no detectable traces of silver ions.

### 4.5. Treatment of Worms with AgNPs

Synchronized N2 and *cua-1^H828Q^* strains were grown in a medium supplemented with 0.25 μg/mL of AgNPs.

### 4.6. Lifespan

Ten adult worms were placed on silver-containing (0.25 and 1 µg/mL) or control NGM plates. The plates were incubated at 20 °C for 1 h. During this time, each nematode laid approximately 50 eggs, after which the worms were removed. The resulting plates were incubated at 20 °C for 3 days. At the onset of the reproductive period, worms were transferred to new plates with appropriate concentrations of AgNPs every two days to avoid contamination by progeny. Surviving worms were counted during the transfer and considered dead when animals failed to respond to several head or tail stimulations. The nematodes were monitored until all worms were considered dead.

### 4.7. Isolation of Plasma Membrane Fraction

Young adult nematodes were cultured in a liquid medium, collected by centrifugation at 1300× *g* for 5 min (Allegra X-30R centrifuge, Beckman Coulter, Brea, CA, USA), and washed several times with M9 buffer. Aliquots of worm samples (~600 μL) were homogenized four times for 30 s using a T 10 basic ULTRA-TURRAX disintegrator (IKA, Staufen im Breisgau, Germany) at a 1:3 *v*/*v* ratio in 0.25 M sucrose prepared in homogenization buffer (HB) containing 10 mM Tris-HCl buffer, 120 mM KCl, 5 mM MgCl_2_, 5 mM dithiothreitol, and 0.5 μg/mL of a cocktail of protease inhibitors (Sigma, St. Louis, MO, USA). The fractions were isolated by differential centrifugation (Figure 5). Briefly, the primary homogenate was centrifuged at 1000× *g* for 10 min. The pellet (P1) was then carefully resuspended in lysis buffer (20 mM Tris-HCl pH = 8, 150 mM NaCl, 0.5% NP-40, 0.5% Triton X-100, 1mM EDTA, 10% glycerol), and the mixture was incubated on ice for 2 h. Under these conditions, only the plasma membrane is solubilized, while the nuclear envelope remains intact. The incubation mixture was then clarified by centrifugation at 2000× *g* for 10 min. The supernatant obtained was used as a fraction containing plasma membrane proteins for further analysis.

### 4.8. Gene Expression Analyses

Synchronized populations of N2 and *cua-1^H828Q^* worms were grown in a liquid medium with *E. coli* OP50 as a food source. Animals were collected at different stages of their life cycle (L1, L2, L3, L4, young adult) and washed several times with M9 buffer to remove bacteria; then, 200 μL of each worm sediment was transferred into 2 mL tubes. Three biological replicates were prepared for each experimental group. Worm samples were disrupted by an ULTRA-TURRAX T 10 disperser (IKA, Staufen im Breisgau, Germany) in 200 μL of TRIzol (TriPure Isolation Reagent, Invitrogen, Waltham, MA, USA) (10 s of disruption, 10 s of pause, 3 cycles, on ice). Then, another 200 μL of TRIzol was added and the procedure was repeated (the total volume of added TRIzol was 1 mL). Total RNA was isolated using phenol–chloroform method and treated with RNase-free DNase I (Thermo Fisher Scientific, Waltham, MA, USA). The integrity of total RNA was assessed by running an aliquot (2000–3000 ng) of each RNA sample on an agarose gel stained with ethidium bromide. A total of 1 μg of total RNA was used for cDNA synthesis (RevertAid First Strand cDNA Synthesis Kit, Thermo Fisher Scientific, Waltham, MA, USA). Real-time PCR was performed with ×5 SYBR Green Real-Time PCR Master Mix (“Evrogen”, Moscow, Russia) using a CFX96 amplificator (Bio-Rad, Hercules, CA, USA) with two technical replicates. Primers were designed using the ”Primer-BLAST“ service (NCBI, Bethesda, MD, USA). The list of specific primer sequences used is provided in the Supplementary (Table S1). Primers were commercially synthesized by “Evrogen” (Moscow, Russia). Fold-change values were calculated using the 2^(−ΔΔCt)^ method, with all values normalized to the geometric mean of *pmp-3* and *cdc-42* expression levels.

### 4.9. Western Blotting (WB)

The standard stages of WB (electrophoresis in PAGE under denaturing (with SDS, 2-mercapto-ethanol and heating at 95 °C) and non-denaturing conditions, the transfer of proteins to a nitrocellulose membrane, the control of protein transfer by Ponceau S, membrane loading with skimmed milk, binding with the primary and secondary antibodies, membrane washes, and the detection of immune complexes) were carried out in full accordance with the procedures described earlier [36]. Specifically, a fraction of plasma membrane proteins (20 µg of protein per lane) was used, and sample equilibration was based on the total protein concentration measured by the Bradford colorimetric assay. IgG from rabbits immunized with electrophoretically pure Cp preparations (A610/280 = 0.047), isolated from human, rat, or mouse blood serum, were used as primary antibodies [37]. Horseradish-peroxidase-conjugated goat anti-rabbit antibodies (Abcam, Cambridge, UK) were used as secondary antibodies. Immune complex visualization was performed using the ChemiDoc System (“Bio-Rad”, Hercules, CA, USA) using the Clarity Western ECL Blotting Substrate (“Bio-Rad”, Hercules, CA, USA).

### 4.10. Determination of Oxidase and Ferroxidase Activity

Oxidase and ferroxidase activities were detected using the assay-in-gel method. The plasma membrane extracts were fractionated using non-denaturing electrophoresis in 6% polyacrylamide gel (PAAG) with 0.1% Triton X-100. Then, the gels were stained with o-dianisidine to reveal the oxidase activity toward aromatic amines [38] or with the Mohr salt–ferrozine system to estimate ferroxidase activity [39].

### 4.11. Atomic Absorption Spectroscopy

Samples, equalized by mass or protein content, were dissolved in pure nitric acid at 50 °C. Silver concentration was measured using atomic absorption spectrometry (AAS) with electrothermal atomization and the Zeeman correction of nonselective absorption using a ZEEnit 650p spectrometer (Analytic Jena) with automatic sampling duplication.

### 4.12. In Silico Analysis

Was carried out on information retrieved from open databases. Amino acid sequences of 10 multicopper oxidases (MCOs) (Table S2) were retrieved from the GenPept protein sequences database (URL: www.ncbi.nlm.nih.gov/protein, accessed on: 10 December 2024) and compared using the BLASTP program [40]. Clustal Omega software (URL: https://www.ebi.ac.uk/jdispatcher/msa/clustalo, accessed on: 16 December 2024) [41] was used for multiple sequence alignment of MCO sequences. The D-I-TASSER tool (zhanggroup.org/D-I-TASSER, accessed on: 20 December 2024) was used to predict the structure and function of the F21D5.3 protein. Multiple pattern alignments were then identified using data from the PDB (www.rcsb.org, accessed on: 20 December 2024). Full-scale structural models were generated by iteratively assembling Monte Carlo simulation fragments under the I-TASSER force field guidance and deep learning contact/distance/VS constraints. Finally, the biological functions of the queried protein were inferred using the structural function annotation method. The PyMol software (www.pymol.org, accessed on: 25 December 2024) was used to visualize the predicted putative structure of F21D5.3. WORMSEQ, a single-cell transcriptional atlas of the wild-type adult *C. elegans* (wormseq.org accessed on: 15 December 2024), was used to visualize the expression of the genes of interest (*chca-1*, *cuc-1*, *cua-1*, and *F21D5.3*) in all cell types. Gene expression values were expressed in scaled transcripts per million (scaled TPM) [42]. The N-GlicoSite tool (www.hiv.lanl.gov accessed on: 15 December 2024) was used for the search of potential N-glycosylation sites.

### 4.13. Statistical Analysis

Data are presented as mean values of three independent experiments ± SD. The statistical method used in each case is indicated in the legend. To determine the E-value, amino acid sequences were analyzed using BLASTP and Clustal Omega software. Amino acid sequences were scored as being significantly similar at an E-value < 0.001. The results of changes in *chca-1*, *cuc-1*, *cua-1*, and *F21D5.3* gene expression levels were analyzed using a one-way ANOVA followed by Tukey’s post hoc test. Differences were considered statistically significant with a *p*-value of less than 0.05.

## Data Availability

The original contributions presented in this study are included in the article. Further inquiries can be directed to the corresponding author(s).

## Supplementary Materials

The following supporting information can be downloaded at: https://www.mdpi.com/article/10.3390/ijms26104776/s1.

## References

[1] Philipp T.M., Gernoth L., Will A., Schwarz M., Ohse V.A., Kipp A.P., Steinbrenner H., Klotz L.-O. (2023). Selenium-binding protein 1 (SELENBP1) is a copper-dependent thiol oxidase. Redox Biol..

[2] Lutsenko S., Roy S., Tsvetkov P. (2025). Mammalian copper homeostasis: Physiological roles and molecular mechanisms. Physiol. Rev..

[3] Jomova K., Alomar S.Y., Nepovimova E., Kuca K., Valko M. (2025). Heavy metals: Toxicity and human health effects. Arch. Toxicol..

[4] Han J. (2023). Copper trafficking systems in cells: Insights into coordination chemistry and toxicity. Dalton Trans..

[5] Vogt S., Ralle M. (2013). Opportunities in multidimensional trace metal imaging: Taking copper-associated disease research to the next level. Anal. Bioanal. Chem..

[6] Chen J., Jiang Y., Shi H., Peng Y., Fan X., Li C. (2020). The molecular mechanisms of copper metabolism and its roles in human diseases. Pflugers Arch..

[7] Abdeen A.H., Trist B.G., Double K.L. (2022). Empirical evidence for biometal dysregulation in Parkinson’s disease from a systematic review and Bradford Hill analysis. NPJ Parkinsons Dis..

[8] Li Y., Han Y., Shu Q., Kan Y.-K., Wang Z. (2025). Cuproptosis and copper as potential mechanisms and intervention targets in Alzheimer’s disease. Biomed. Pharmacother..

[9] Xue Q., Kang R., Klionsky D.J., Tang D., Liu J., Chen X. (2023). Copper metabolism in cell death and autophagy. Autophagy.

[10] Eljazzar S., Abu-Hijleh H., Alkhatib D., Sokary S., Ismail S., Al-Jayyousi G.F., Tayyem R. (2023). The Role of Copper Intake in the Development and Management of Type 2 Diabetes: A Systematic Review. Nutrients.

[11] Incecik F., Bisgin A., Yılmaz M. (2018). MEDNIK syndrome with a frame shift causing mutation in AP1S1 gene and literature review of the clinical features. Metab. Brain Dis..

[12] Blades B., Ayton S., Hung Y.H., Bush A.I., La Fontaine S. (2021). Copper and lipid metabolism: A reciprocal relationship. Biochim. Biophys. Acta Gen. Subj..

[13] Valko M., Rhodes C.J., Moncol J., Izakovic M., Mazur M. (2006). Free radicals, metals and antioxidants in oxidative stress-induced cancer. Chem. Biol. Interact..

[14] Harada M. (2004). Wilson disease and hepatocellular carcinoma. Intern. Med..

[15] Petruzzelli R., Catalano F., Crispino R., Polishchuk E.V., Elia M., Masone A., Lavigna G., Grasso A., Battipaglia M., Sepe L.V. (2025). Prion protein promotes copper toxicity in Wilson disease. Nat. Commun..

[16] Polishchuk E.V., Merolla A., Lichtmannegger J., Romano A., Indrieri A., Ilyechova E.Y., Concilli M., De Cegli R., Crispino R., Mariniello M. (2019). Activation of Autophagy, Observed in Liver Tissues from Patients with Wilson Disease and From ATP7B-Deficient Animals, Protects Hepatocytes from Copper-Induced Apoptosis. Gastroenterology.

[17] Vonk W.I., Wijmenga C., van de Sluis B. (2008). Relevance of animal models for understanding mammalian copper homeostasis. Am. J. Clin. Nutr..

[18] Zischka H., Lichtmannegger J. (2014). Pathological mitochondrial copper overload in livers of Wilson’s disease patients and related animal models. Ann. N. Y. Acad. Sci..

[19] Reed E., Lutsenko S., Bandmann O. (2018). Animal models of Wilson disease. J. Neurochem..

[20] Philipp T.M., Gong W., Köhnlein K., Ohse V.A., Müller F.I., Priebs J., Steinbrenner H., Klotz L.O. (2022). SEMO-1, a novel methanethiol oxidase in Caenorhabditis elegans, is a pro-aging factor conferring selective stress resistance. Biofactors.

[21] Ohse V.A., Klotz L.O., Priebs J. (2024). Copper Homeostasis in the Model Organism *C. elegans*. Cells.

[22] Członkowska A., Litwin T., Dusek P., Ferenci P., Lutsenko S., Medici V., Rybakowski J.K., Weiss K.H., Schilsky M.L. (2018). Wilson disease. Nat. Rev. Dis. Primers.

[23] Chun H., Sharma A.K., Lee J., Chan J., Jia S., Kim B.-E. (2017). The Intestinal Copper Exporter CUA-1 Is Required for Systemic Copper Homeostasis in Caenorhabditis elegans. J. Biol. Chem..

[24] Catalano F., O’Brien T.J., Mekhova A.A., Sepe L.V., Elia M., De Cegli R., Gallotta I., Santonicola P., Zampi G., Ilyechova E.Y. (2024). A new Caenorhabditis elegans model to study copper toxicity in Wilson disease. Traffic.

[25] Ovchinnikova E.V., Garbuz M.M., Ovchinnikova A.A., Kumeiko V.V. (2024). Epidemiology of Wilson’s Disease and Pathogenic Variants of the ATP7B Gene Leading to Diversified Protein Disfunctions. Int. J. Mol. Sci..

[26] Vasin A., Klotchenko S., Puchkova L. (2013). Phylogenetic analysis of six-domain multi-copper blue proteins. PLoS Curr..

[27] Skvortsov A.N., Ilyechova E.Y., Puchkova L.V. (2023). Chemical background of silver nanoparticles interfering with mammalian copper metabolism. J. Hazard Mater..

[28] Jimenez-Arroyo N., Rudino-Pinera E. (2016). Crystal Structure of Laccase from Thermus Thermophilus HB27 Complexed with Ag, Crystal of the Holoenzyme Soaked for 30 m in 5 mM AgNO_3_ at 278 K.

[29] Orlov I.A., Sankova T.P., Babich P.S., Sosnin I.M., Ilyechova E.Y., A Kirilenko D., Brunkov P.N., Ataev G.L., E Romanov A., Puchkova L.V. (2016). New silver nanoparticles induce apoptosis-like process in E. coli and interfere with mammalian copper metabolism. Int. J. Nanomed..

[30] Ilyechova E.Y., Saveliev A.N., Skvortsov A.N., Babich P.S., Zatulovskaia Y.A., Pliss M.G., Korzhevskii D.E., Tsymbalenko N.V., Puchkova L.V. (2014). The effects of silver ions on copper metabolism in rats. Metallomics.

[31] Sankova T.P., Sosnin I.M., Karpenko M.N., Ilyechova E.Y., Orlov Y.A., Polyakov D.S., Skomorokhova E.A., Sukhanova A.S., Rozhkova N.A., Babich P.S. (2015). On the biological activity of silver nanoparticles. Mater. Phys. Mech..

[32] Skomorokhova E.A., Sankova T.P., Orlov I.A., Savelev A.N., Magazenkova D.N., Pliss M.G., Skvortsov A.N., Sosnin I.M., A Kirilenko D., Grishchuk I.V. (2020). Size-Dependent Bioactivity of Silver Nanoparticles: Antibacterial Properties, Influence on Copper Status in Mice, and Whole-Body Turnover. Nanotechnol. Sci. Appl..

[33] Babich P.S., Skvortsov A.N., Rusconi P., Tsymbalenko N.V., Mutanen M., Puchkova L.V., Broggini M. (2013). Non-hepatic tumors change the activity of genes encoding copper trafficking proteins in the liver. Cancer Biol Ther..

[34] Patel D., Xu C., Nagarajan S., Liu Z., O Hemphill W., Shi R., Uversky V.N., A Caldwell G., A Caldwell K., Witt S.N. (2018). Alpha-synuclein inhibits Snx3-retromer-mediated retrograde recycling of iron transporters in S. cerevisiae and *C. elegans* models of Parkinson’s disease. Hum. Mol. Genet..

[35] Weishaupt A.K., Lamann K., Tallarek E., Pezacki A.T., Matier C.D., Schwerdtle T., Aschner M., Chang C.J., Stürzenbaum S.R., Bornhorst J. (2024). Dysfunction in atox-1 and ceruloplasmin alters labile Cu levels and consequently Cu homeostasis in C. elegans. Front. Mol. Biosci..

[36] Magazenkova D.N., Skomorokhova E.A., Farroukh M.A., Zharkova M.S., Jassem Z.M., Rekina V.E., Shamova O.V., Puchkova L.V., Ilyechova E.Y. (2013). Influence of Silver Nanoparticles on the Growth of Ascitic and Solid Ehrlich Adenocarcinoma: Focus on Copper Metabolism. Pharmaceutics.

[37] Sokolov A.V., Kostevich V.A., Romanico D.N., Zakharova E.T., Vasilyev V.B. (2012). Two-Stage Method for Purification of Ceruloplas min Based on Its Interaction with Neomycin. Biochemistry.

[38] Owen J., Smith H. (1961). Detection of ceruloplasmin after zone electrophoresis. Clin. Chim. Acta.

[39] Chen H., Attieh Z.K., Su T., Syed B.A., Gao H., Alaeddine R.M., Fox T.C., Usta J., Naylor C.E., Evans R.W. (2004). Hephaestin is a ferroxidase that maintains partial activity in sex-linked anemia mice. Blood.

[40] Altschul S.F., Gish W., Miller W., Myers E.W., Lipman D.J. (1990). Basic local alignment search tool. J. Mol. Biol..

[41] Madeira F., Pearce M., Tivey A.R.N., Basutkar P., Lee J., Edbali O., Madhusoodanan N., Kolesnikov A., Lopez R. (2022). Search and sequence analysis tools services from EMBL-EBI in 2022. Nucleic Acids Res..

[42] Packer J.S., Zhu Q., Huynh C., Sivaramakrishnan P., Preston E., Dueck H., Stefanik D., Tan K., Trapnell C., Kim J. (2019). A lineage-resolved molecular atlas of C. elegans embryogenesis at single-cell resolution. Science.

