# Supplementary material for: Some Properties of the C. elegans Multicopper Oxidase F21D5.3, an Ortholog of Human Ceruloplasmin
Source: Int J Mol Sci. 2025 May 16;26(10):4776. doi: 10.3390/ijms26104776 (PMC12112694; doi:10.3390/ijms26104776)
Supplement: Supplementary file 1 [file ijms-26-04776-s001.zip › ijms-3577468-supplementary.pdf]

Supplementary Materials

Some Properties of the *C. elegans* Multicopper Oxidase F21D5.3, an Ortholog of Human Ceruloplasmin

Polina D. Samuseva[1,2,#], Aleksandra A. Mekhova-Caramalac[3,#], Federico Catalano[4], Anna D. Shchukina[1], Sofia A. Baikina[3], Daria N. Magazenkova[1,2,3], Ludmila V. Puchkova[1,2,3] and Ekaterina Yu. Ilyechova[1,2,3,*]

1 Research Center of Advanced Functional Materials and Laser Communication Systems, ADTS Institute, ITMO University, 197101 St. Petersburg, Russia
2 Laboratory of Biochemical Genetics, Research Institute of Experimental Medicine, 197022 St. Petersburg, Russia
3 Institute of Biomedical Systems and Biotechnology, Peter the Great St. Petersburg Polytechnic University, 195251 St. Petersburg, Russia
4 Institute of Biosciences and BioResources, National Research Council (CNR), 80131 Naples, Italy

* Correspondence: ilichevaey@itmo.ru; Tel.: +7-921-760-5274

Table S1. The amino acid sequences of MCOs were used in the bioinformatics analysis.

Organism	Sequence number	Abbreviations
Caenorhabditis elegans	NP_001255321.1	F21D5.3_CAEEL
Mus musculus (Mouse)	NP_001263177.1	CERU_MOUSE
Mus musculus (Mouse)	NP_001153099.1	HEPH_MOUSE
Rattus norvegicus (Rat)	AAA40917.1	CERU_RAT
Saccharomyces cerevisiae	NP_013774.1	FET3_YEAST
Salmonella typhimurium	WP_000946047.1	CUEO_SALTY
Cucurbita pepo var. melopepo (Zucchini)	A51027	ASO_CUCPM
Homo sapiens (Human)	NP_000087.1	CERU_HUMAN
Homo sapiens (Human)	NP_001124332.1	HEPH_HUMAN
Homo sapiens (Human)	NP_001092142.1	HPHL1_HUMAN

Table S2. Sequences of primers used to determine the concentration of mature transcription products.

Gene	Forward primer, 5′→3′	Reverse primer, 5′→3′
F21D5.3	AGG GAA CTC ATT GGT ATC ACG G	CCA ACA TCC GTT GAC ACT GG
cdc-42	GTT TGC TTC TCC GTG GTT GC	CCA ACA TCC GTT GAC ACT GG
pmp-3	GTT CCC GTG TTC ATC ACT CAT	ACA CCG TCG AGA AGC TGT AGA

Abbreviation: *F21D5.3* – nematode putative MMO (Tm, ℃, 64.3 (Forward primer) and 63.9 (Reverse primer)); *cdc-42* – guanine nucleotide exchange factor (Tm, ℃, 64.6 (Forward primer) and 63.6 (Reverse primer)); *pmp-3* – ABC transporter (Tm, ℃, 63 (Forward primer) and 65.3 (Reverse primer)).

```
CERU_HUMAN      QVQECNKS-      341
F21D5.3         IVVDQNDTS      220
                 * : *.:
```

Figure S1. Homologous N-glycosylation sites of human Cp and F21D5.3

	Description	Max Score	Total Score	Query Cover	E value	Per. Ident	Acc. Len	Accession
☑	FET2_YEAST	95.1	148	52%	9e-24	29.04%	636	Query_3514743
☑	CERU_MOUSE	32.7	61.2	8%	2e-04	34.88%	1061	Query_3514741
☑	CERU_HUMAN	31.2	57.8	7%	7e-04	32.56%	1065	Query_3514742

Figure S2. Sequence similarity of F21D5.3 with human and mouse Cp, and yeast ferroxidase Fet3. Protein sequence alignment was performed using the BLASTP algorithm.
